# Green Innovation in Regional Logistics: Level Evaluation and Spatial Analysis

**DOI:** 10.3390/ijerph20010735

**Published:** 2022-12-30

**Authors:** Hao Zhang, Xin Sun, Kailong Dong, Lianghui Sui, Min Wang, Qiong Hong

**Affiliations:** 1 Jiangsu Key Laboratory of Traffic and Transportation Security, Huaiyin Institute of Technology, Huai’an 223003, China; 2 School of Traffic Engineering, Huaiyin Institute of Technology, Huai’an 223003, China; 3 School of Computer and Software Engineering, Huaiyin Institute of Technology, Huai’an 223003, China; 4 School of Transportation Engineering, Nanjing Tech University, Nanjing 211816, China; 5 Business School, Jiangsu Vocational College of Electronics and Information, Huai’an 223003, China

**Keywords:** logistics industry, green innovation level, index system, game theory, GRA-TOPSIS, spatial distribution

## Abstract

Green innovation is imperative for the high-quality and sustainable development of the modern logistics industry. It is also key for achieving the goals of peak emissions and carbon neutrality. This study provides a way of thinking about the evaluation of the green innovation level of the logistics industry. The variance inflation factor-variance coefficient method was employed to construct an evaluation index system of the regional logistics green innovation level (RLGIL) from three dimensions. Empirical data were collected from statistical yearbooks covering 30 provinces in China from 2013 to 2017. Thereafter, the combination weighting-based GRA-TOPSIS method was applied to evaluate the RLGIL, and the spatial distribution differences and spatiotemporal evolution characteristics of inter-provincial green innovation levels were analyzed. The RLGILs in the 30 provinces were found to be generally unbalanced, and the differences between the eastern and western regions were significant. Guangdong, Jiangsu, and Zhejiang had stronger RLGILs, whereas most other provinces did not reach the average level. The RLGIL of the 30 provinces had a high positive spatial correlation and spatial aggregating effect. From a national perspective, the values for the RLGIL were generally higher in the eastern and southern regions and lower in the western and northern regions. Although significant differences were found in the RLGIL of these provinces, the overall development trend was stable.

## 1. Introduction

Environmental pollution has become a serious challenge for global economic development. Under the framework of the Paris Climate Change Agreement, the Chinese government has proposed that peak carbon dioxide emissions should be reached by 2030 and that carbon neutrality should be achieved by 2060. However, the extensive growth of the logistics industry in China is accompanied by high energy expenditure, serious waste of resources, and heavy environmental burden [1,2]. To achieve energy savings and green economic development, the National 14th Five-Year Plan (2021–2025) emphasizes the urgent need to establish a green, low-carbon, and circular economic system by promoting modern logistics technology and green innovations.

Green innovation is to achieve coordinated development of economy, resources, and environment while reducing environmental pollution and resource consumption [3]. At present, a large number of green innovative products and integrated application practices have emerged in the logistics industry, such as new energy distribution vehicles, electronic sheets, shared express boxes, green warehousing, and network freight platforms, which effectively reduce the consumption of resources and influence on the environment. Although some achievements have been made, there is still much room for the overall green innovation level in China’s logistics industry, especially for a large number of small and medium-sized logistics enterprises and regional differences [4]. Therefore, many scholars have conducted a large number of studies on the influencing factors of green innovation, green innovation efficiency, green innovation performance, green innovation ability, etc. The scope of the studies includes regions, industries, enterprises, etc, [5,6]. However, most of the research objects are manufacturing enterprises. Although the logistics industry does not directly produce products, its impact on resources and the environment cannot be ignored.

Many scholars have evaluated green innovation efficiency, performance, and ability from different dimensions using single or combined evaluation methods. However, the connotative definitions of these terms, such as green innovation efficiency, green innovation performance, and green innovation ability, are still ambiguous. In addition, the selection of the evaluation index is highly subjective. A single evaluation method may lead to inconsistent results with research objectives [4,7,8,9,10,11,12]. As a result, the conclusions have neither been comparable nor stable. To better guide green innovation practices and decisions, this study sought to discuss the use of the following terminology from the literature:

- Green Innovation Efficiency

Green innovation efficiency refers to the general term for the use of technological innovation and related means to reduce resource consumption, improve the environment, and protect the ecology to obtain the greatest possible economic, environmental, and ecological benefits [13]. In contrast to traditional innovation efficiency, scholars have defined green innovation efficiency as the integration of environmental outputs and economic benefits by considering the inputs and outputs of innovation resources [14,15]. Regarding evaluation methods to measure green innovation efficiency, the existing literature usually employs two approaches. One approach is based on the enterprise’s microscopic data and uses principal component analysis, factor analysis, and structural equation modeling. Fang et al. constructed a non-radial distance function-data envelopment analysis (DDF-DEA) three-stage green innovation efficiency evaluation model to measure the green innovation efficiency of China’s heavily polluting industries [16]. Long et al. evaluated the efficiency of green innovation in 30 provinces of China by overcoming the shortcomings of the radial model and slack-based metric (SBM) using the ϵ-based metric (EBM) global Malmquist–Luenberger (ML) model [17]. The other approach is based on macroscopic statistical data and adopts a single factor, integrated factors, and total factor productivity [7,9,10,18,19].

- Green Innovation Performance

Green innovation performance refers to the performance evaluation of the process of adding value to the economic and social value of enterprises by developing or adopting new technologies for green innovation activities [20]. According to Huang [21], green innovation performance reflects changes in green processes and products/services in business operations. Cheng [22] proposed that green innovation performance is an outcome of the sustainability orientation possessed by diversifying green entrants. Focusing on the perspectives of static efficiency and dynamic productivity, Yang et al. empirically analyzed the impact of manufacturing intelligence on green innovation performance using the dynamic spatial lag model (DSAR), mediation effect model, and moderation effect model [23]. The relationship between green innovation and firm performance, which includes economic performance, environmental performance, and competitive advantage, was explored in existing literature [24,25,26,27,28]. These studies mainly focused on the static perspective to measure green innovation performance and its influencing factors.

- Green Innovation Ability

Green innovation ability refers to the ability of an enterprise to introduce green technology into the whole process of technological innovation when promoting the coordinated development of the economy and environment, and to achieve a certain standard for green technological innovation in a certain period of time in the future [29]. From a resource-based view, some scholars believe that green innovation ability is the technical innovation ability of enterprises or industries to implement energy conservation and emission reduction or produce green products [30,31]. Xu and Zhai [6] selected 14 indices from the dimensions of innovation environment, input, and output to construct an evaluation index system. The cloud model, entropy method, and DEMATEL method are used to determine the index weight to evaluate the green innovation ability of manufacturing enterprises in the innovation network. Most relevant studies established an evaluation system to measure the potential or capacity of green innovation from the perspective of input and output [32]. Yu et al.’s study of dynamic capabilities and stakeholder theory examines the moderating role of environmental dynamics and big data analytics capabilities in the link between green dynamic capabilities and green innovation [33].

Although a unified standard does not exist for the measurement of the green innovation level and the terminologies remain ambiguous, these existing results provide rich and high-quality insights into the evaluation and spatial-temporal evolution characteristics analysis of the green innovation level in the regional logistics industry.

This study aimed to take a further step by addressing the following research gaps: (1) The research objects of green innovation evaluation are mainly manufacturing enterprises, urban business districts, high-tech enterprises, etc., and studies on the green innovation level of regional logistics have not garnered sufficient attention. (2) In the selection of an evaluation index, some empirical studies have adopted a single index or redundant index, and the strong subjectivity of index selection depends on experience and the neglect of the influence of external environmental factors. (3) Existing index weighting methods cannot deal with data attribute distortion and unreasonable index weights. Meanwhile, current TOPSIS evaluation methods tend to ignore the correlation between indices. Further, the evaluation object is often close to the positive/negative ideal solutions. (4) The green innovation level is dynamically changing, and existing studies cannot reflect the spatial-temporal dynamic and evolutionary characteristics of the RLGIL.

To fill the research gaps, this study sought to add the environmental level dimension to the two traditional dimensions of input and output. In addition, an RLGIL evaluation index system was established from the perspective of the basic elements supporting green innovation. Thereafter, a methodological framework was built based on index screening, comprehensive evaluation, and spatial-temporal evolution. First, the variance inflation factor (VIF)-variation coefficient method was used to refine the index system to avoid the blindness of index selection. Second, the combination weighting method based on game theory was used to weigh the evaluation index, and the GRA-TOPSIS method was applied to measure the green innovation level of logistics in 30 provinces of China. Third, using the Jenks natural breaks classification method with the global and local Moran’s I indices, the spatial distribution differences and spatial-temporal evolution characteristics of inter-provincial green innovation levels were analyzed, and countermeasures to promote the logistics industry’s green innovation level were finally proposed.

## 2. Construction of the Evaluation Index System of Green Innovation Level

To solve the problems of information redundancy, subjectivity, and neglect of external environmental factors in existing research, this study proposed an index screening model based on the VIF variation coefficient method by referring to the determination method of the evaluation system in the literature [34,35,36]. The VIF in the model can test the multicollinearity problem in the index, and the variation coefficient method can select an index with large information content.

### 2.1. Primary Selection of the Evaluation Index

Based on the relevant achievements of green innovation and logistics innovation [25,31,35], an evaluation index system comprising the green innovation input level (GIIL), green innovation output level (GIOL), and green innovation environment level (GIEL) was constructed. Green innovation in the logistics industry is inseparable from human and financial investment. The two inputs can promote the continuous improvement of the logistics green innovation system in various regions. Similarly, the continuous improvement of the logistics green innovation system will also drive employment and economic development in various regions. Therefore, the GIIL includes two first-level indices of human resource input and financial fund input. Secondly, there is a close relationship between green innovation in the logistics industry and the desired output and non-expected output, which is a key factor to test the level of innovation ability. The expected output of the logistics industry is the continuous driving force for maintaining green innovation and competition in the logistics industry, and the emission reduction of undesired output can also improve the level of green innovation in the logistics industry. Therefore, The GIOL includes two first-level indices: expected output and non-expected output. The external environment of the region is the basic guarantee for the green innovation of the logistics industry. The development of the social economy provides the driving force for the development of green innovation in regional logistics. Good logistics infrastructure conditions and informatization conditions can smoothly carry out the logistics green innovation activities. Therefore, the GIEL includes three first-level indices: social development, logistics infrastructure, and informational development. A preliminary evaluation index system was selected based on the above indices, as shown in Table 1.

### 2.2. Index Screening Based on the VIF-Variance Coefficient Method

#### 2.2.1. Index Screening Based on the Multicollinearity Test

First, in each index layer, regression was performed on index variable *i* and explanatory variables besides *i*. The coefficient of determination $R_{i}^{2}$ of the index variable *i* was used to reflect the correlation between the index variable *i* and other indices. A smaller $R_{i}^{2}$ value indicates that the information reflected by index *i* is different from that of other indices and should be retained; similarly, a larger $R_{i}^{2}$ value indicates that the information reflected by index *i* can be replaced by that of other indices, and index *i* should be deleted. Finally, the VIF of the index variable *i* (${\mathrm{VIF}}_{i}$) was calculated to determine whether multicollinearity exists between index *i* and other indices and eliminate redundant information between indices. If ${\mathrm{VIF}}_{i}>10$, a multicollinearity exists between index *i* and other indices, and the index variable *i* should be deleted [37]. The formula for the coefficient of determination and variance inflation factor of index *i* is expressed as follows:

(1) $$R_{i}^{2}=\frac{\sum_{j=1}^{n}{\left({\hat{x}}_{ij}-{\bar{x}}_{i}\right)}^{2}}{\sum_{j=1}^{n}{\left(x_{ij}-{\bar{x}}_{i}\right)}^{2}}$$

(2) $${\mathrm{VIF}}_{i}=\frac{1}{1-R_{i}^{2}}$$

where $R_{i}^{2}$ is the coefficient of determination of index $i,{\bar{x}}_{i}$ is the mean value of index $i,x_{ij}$ is the original value of the *i* index in the *j* region, ${\hat{x}}_{ij}$ is the regression fit value of index *i* in region *j*, and ${\mathrm{VTF}}_{i}$ is the variance inflation factor of index *i*.

#### 2.2.2. Index Screening Based on the Variation Coefficient Method

The coefficient of variation $v_{i}$ of indicator *i* was calculated to reveal the amount of information that can be represented by the indicator. A larger value of $v_{i}$ indicates that indicator *i* contains more information and is more representative. Conversely, indicator *i* contains less information.

The mean value of the coefficient of variation of all indices in the index layer was used as the judgment criterion, and indices less than or equal to the mean value were deleted to filter out more representative indices. The formula for the coefficient of variation $v_{i}$ is expressed as follows:

(3) $$v_{i}=\frac{\sqrt{\frac{1}{n}\sum_{j=1}^{n}{\left(x_{ij}-\bar{x_{i}}\right)}^{2}}}{\bar{x_{i}}}$$

where *n* is the number of provinces evaluated, ${\bar{x}}_{i}$ is the mean value of the data of the *i*-th index of each province, and $x_{ij}$ is the value of the *i*th indicator of the j-th province.

#### 2.2.3. Rationality Judgment of Evaluation Index System after Screening

The information contribution rate of the selected index system was calculated. The judgment criterion for evaluating the reasonableness of the index system was to reflect more than 85% of the original information with less than 35% of the original indices [34], ultimately screening out the final evaluation index system:

(4) $$\;\mathrm{In}\;=\mathrm{tr}\left(S_{s}\right)/\mathrm{tr}\left(S_{h}\right)$$

where *S* represents the covariance matrix of the index matrix, $tr(S_{s})$ represents the trace of the covariance matrix, *s* represents the number of filtered indices, and *h* represents the number of the original indices.

### 2.3. Establishment of the Evaluation Index System

The data of the index sets from 30 provinces in China were selected for index screening based on the VIF-variance coefficient method. As the indices of the “non-expected output” are few and not easy to measure for the logistics industry, carbon dioxide emissions that can be measured in the logistics industry were selected as the representative for the calculation, and 21 secondary indices were retained. The trace of the covariance of the screened GIOL and trace of the initial index covariance were calculated, and the information contribution rate was 91.51%. Similarly, the information contribution rates of the screened GIOL and GIEL indices were 85.69% and 86.34%, respectively, which satisfied the judgment criteria for evaluating the reasonableness of the index system. Table 2 presents the filtered evaluation index system.

## 3. Research Methods

**Figure 1:**
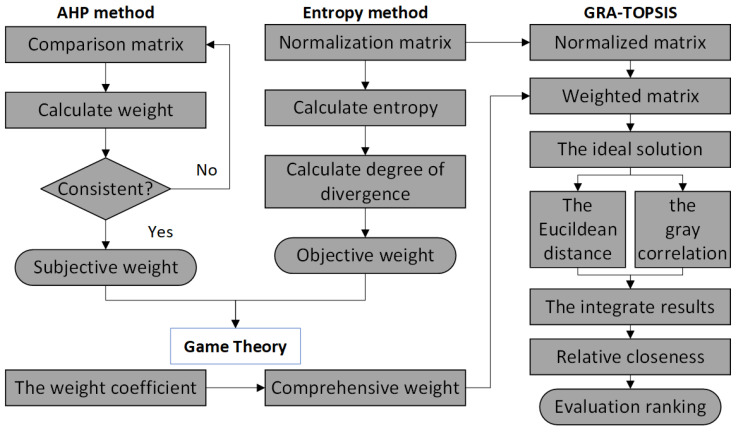
GRA-TOPSIS method flow chart.

This study combines the AHP method, entropy method, game theory, grey relational degree method, and TOPSIS method to form the GRA-TOPSIS method [38,39] to rank the RLGILs. Aiming at the problems of data attribute distortion and unreasonable index weight in the existing index weighting methods, this paper uses game theory to carry out combination weighting. For the limitations of the TOPSIS method, such as ignoring the correlation between indicators and the occurrence of evaluation objects close to both positive and negative ideal points, a combined evaluation model based on GRA-TOPSIS is used [40]. The model not only considers the actual Euclidean distance of the evaluation object in the multidimensional space, but also fully considers the correlation degree among the indicators. Firstly, the subjective weight is calculated by the AHP method, and the objective weight is calculated by the entropy method. Finally, we use game theory to obtain optimal weights based on subjective weights and objective weights and use the optimal weights for the GRA-TOPSIS method. The flow chart is shown in Figure 1.

### 3.1. Combination Weighting Method Based on the Game Theory

#### 3.1.1. AHP Method

The most widely used method of subjective weight analysis is the analytic hierarchy process (AHP). This method compares and scores the importance of each factor through the willingness of decision makers. The higher the importance, the higher the comparison score. By comparing each factor in pairs, scoring to determine the judgment matrix, and by calculating the consistency factor CR of the matrix, we judge whether it passes the consistency test [29]. The weight results that pass the consistency test can be considered reasonable, otherwise, the judgment matrix needs to be readjusted until it passes the test.

#### 3.1.2. Entropy Method

The entropy weight method is a method of objective assignment, which can reflect the degree of variability in standard datasets. The greater the degree of variability, the higher the weight of this criterion, and vice versa [41].

To determine the index weight by the entropy weight method, it is necessary to normalize the original data of the index first.

(1) The normalization matrix *X* and the normalization formula are as follows:

(5) $$X={\left\{x_{ij}\right\}}_{m\times n}=\left[\begin{array}{cccc} x_{11} & x_{12} & \ldots & x_{1n}\\ x_{21} & x_{22} & \ldots & x_{2n}\\ \vdots & \vdots & \ddots & \vdots \\ x_{m1} & x_{m2} & \ldots & x_{mn} \end{array}\right]\left(i=1,2,\ldots ,m,j=1,2,\ldots ,n\right),$$

(6) $$x_{ij}=\frac{b_{ij}}{\sum_{i=1}^{m}b_{ij}}\left(i=1,2\ldots m,j=1,2,\ldots ,n\right)$$

where $b_{ij}$ is the original value and $x_{ij}$ is the normalized value.

(2) Calculate the entropy value of the jth index:

(7) $$e_{j}=-\frac{1}{\ln n}\sum_{i=1}^{m}\left(x_{ij}\ln x_{ij}\right)\left(j=1,2,\ldots ,n\right)$$

(3) Calculate the degree of divergence $z_{j}$:

(8) $$z_{j}=1-e_{j}$$

(4) Determine the weight $W_{j}$ of each evaluation index:

(9) $$W_{j}=\frac{z_{j}}{\sum_{j=1}^{n}z_{j}}\left(j=1,2,\ldots ,n\right)$$

#### 3.1.3. The Game Theory

The principle of the combination weighting method under game theory [42,43] is to treat the subjective weights of indicators determined by the hierarchical analysis method as one side of the game, and the objective weights of indicators determined by the entropy weighting [41] method as the other side of the game, then the optimal combination weight is the combination weight in the equilibrium state reached by both sides of the game [44]. From the point of view of mathematical principles, both sides of the game should satisfy the objective weight and subjective weight in the equilibrium state to achieve the minimum sum of deviations between them and the combined weight, which is achieved as follows:

(1) A basic set of weight vectors $W=\left\{w_{1},w_{2},\ldots ,w_{n}\right\}$ consists of *n* weight vectors. A possible set of weights consists of any linear combination of *n* vectors, expressed as:

(10) $$W=\sum_{k=1}^{n}\lambda_{k}w_{k}^{T}\left(\lambda_{k}>0\right)$$

where *w* represents a possible weight vector in set *W*, and $\lambda =\left(\lambda_{1},\lambda_{2},\ldots ,\lambda_{n}\right)$ is the weight coefficient.

(2) The objective function was established to seek the optimal linear combination coefficients $\lambda_{k}$ to minimize the sum of the deviations between the combination weights *w* and $w_{k}$, at which time the combination weights are the optimal combination weights $W^{*}$. The objective function is as follows:

(11) $$\min{∥\sum_{k=1}^{n}\lambda_{k}w_{k}^{T}-w_{i}^{T}∥}_{2}\left(i=1,2,\ldots ,n\right)$$

(3) The optimal first-order derivative condition of Equation (11) is shown in Equation (12), based on the differentiation property of the matrix [39,42,43,44,45]:

(12) $$\sum_{k=1}^{n}\lambda_{k}w_{i}w_{k}^{T}=w_{i}w_{i}^{T}\left(i=1,2,\ldots ,n\right)$$

Equation (12) can be converted into a system of linear equations as shown in Equation (13).

(13) $$\left[\begin{array}{cccc} w_{1}w_{1}^{T} & w_{1}w_{2}^{T} & \ldots & w_{1}w_{n}^{T}\\ w_{2}w_{1}^{T} & w_{2}w_{2}^{T} & \ldots & w_{2}w_{n}^{T}\\ \vdots & \vdots & \ddots & \vdots \\ w_{n}w_{1}^{T} & w_{n}w_{2}^{T} & \ldots & w_{n}w_{n}^{T} \end{array}\right]\left[\begin{array}{c} \lambda_{1}\\ \lambda_{2}\\ \vdots \\ \lambda_{n} \end{array}\right]=\left[\begin{array}{c} w_{1}w_{1}^{T}\\ w_{2}w_{2}^{T}\\ \vdots \\ w_{n}w_{n}^{T} \end{array}\right]$$

The weight coefficients λ are calculated according to Equation (13), and normalized by Equation (14) as:

(14) $$\lambda_{k}^{*}=\frac{\lambda_{k}}{\sum_{k=1}^{n}\lambda_{k}}$$

Finally, the optimal combination weight $W^{*}$ of the evaluation index is calculated as follows:

(15) $$W^{*}=\sum_{k=1}^{n}\lambda_{k}^{*}\cdot w_{k}^{T}$$

### 3.2. Evaluation Model of RLGIL Based on GRA-TOPSIS

A GRA-TOPSIS-based evaluation model was constructed by considering a combination of the two evaluation methods, taking into account the distance and correlation of the alternatives [39,40,46]. The specific implementation steps are as follows:

(1) Calculate the normalized weighted decision matrix $A={\left(A_{ij}\right)}_{m\times n}$, where the weights required to construct the normalized matrix are the combination weights $W^{*}$ determined above:

(16) $$A={\left(A_{ij}\right)}_{m\times n}=W^{*}X_{m\times n}$$

(2) Calculate the positive ideal solution $A^{+}$ and negative ideal solution $A^{-}$ of the determinant matrix:

(17) $$A^{+}=\left(A_{1}^{+},A_{2}^{+},\cdots ,A_{n}^{+}\right),\mathrm{where}\;A_{j}^{+}=\max_{j}\left(A_{ij}\right)\left(i=1,2,\ldots ,m;j=1,2,\ldots ,n\right)$$

(18) $$A^{-}=\left(A_{1}^{-},A_{2}^{-},\cdots ,A_{n}^{-}\right),\mathrm{where}\;A_{j}^{-}=\max_{j}\left(A_{ij}\right)\left(i=1,2,\ldots ,m;j=1,2,\ldots ,n\right)$$

(3) Compute the Euclidean distances $d^{+}$ and $d^{-}$ for the positive and negative ideal solutions:

(19) $$d_{i}^{+}=\sqrt{\sum_{j=1}^{n}{\left(A_{j}^{+}-A_{ij}\right)}^{2}}\left(i=1,2,\ldots ,m\right)$$

(20) $$d_{i}^{-}=\sqrt{\sum_{j=1}^{n}{\left(A_{j}^{-}-A_{ij}\right)}^{2}}\left(i=1,2,\ldots ,m\right)$$

(4) Calculate the gray correlation coefficient matrix $R^{+}$ and $R^{-}$:

(21) $$R^{+}={\left(r_{ij}^{+}\right)}_{m\times n},r_{ij}^{+}=\frac{\min_{i}\min_{j}\left|A_{j}^{+}-A_{ij}\right|+\rho \max_{i}\max_{j}\left|A_{j}^{+}-A_{ij}\right|}{\left|A_{j}^{+}-A_{ij}\right|+\rho \max_{i}\max_{j}\left|A_{j}^{+}-A_{ij}\right|}$$

(22) $$R^{-}={\left(r_{ij}^{-}\right)}_{m\times n},r_{ij}^{-}=\frac{\min_{i}\min_{j}\left|A_{j}^{-}-A_{ij}\right|+\rho \max_{i}\max_{j}\left|A_{j}^{-}-A_{ij}\right|}{\left|A_{j}^{-}-A_{ij}\right|+\rho \max_{i}\max_{j}\left|A_{j}^{-}-A_{ij}\right|}$$

where ρ is the resolution coefficient, 0 < ρ < 1. Generally, ρ = 0.5 is employed.

(5) Calculate the gray correlation $r_{i}^{+}$ and $r_{i}^{-}$ between the evaluation object and the positive and negative ideal solutions:

(23) $$r_{i}^{+}=\frac{1}{n}\sum_{j=1}^{n}r_{ij}^{+}\left(i=1,2,\ldots ,m\right)$$

(24) $$r_{i}^{-}=\frac{1}{n}\sum_{j=1}^{n}r_{ij}^{-}\left(i=1,2,\ldots ,m\right)$$

(6) Calculate the proximity between the evaluation object and the ideal solution and dimensionlessly process the Euclidean distance and correlation:

(25) $$T_{i}=\frac{t_{i}}{\max\left(t_{i}\right)}\left(i=1,2,\ldots ,m\right)$$

when $t_{i}=d_{i}^{+},d_{i}^{-},r_{i}^{+},r_{i}^{-},T_{i}=D_{i}^{+},D_{i}^{-},R_{i}^{+},R_{i}^{-}$.

(7) Merge the Euclidean distance and correlation. When the values of $D_{i}^{+}$ and $R_{i}^{-}$ are larger, the evaluation object is farther away from the negative ideal solution and has a greater correlation with the positive ideal solution. Further, the evaluation object is closer to the ideal solution. When the values of $D_{i}^{-}$ and $R_{i}^{+}$ are larger, the evaluation object is farther away from the positive ideal solution and has a greater correlation with the negative ideal solution. Moreover, the evaluation object deviates from the positive ideal solution:

(26) $$S_{i}^{+}=\alpha D_{i}^{+}+\beta R_{i}^{-}\left(i=1,2,\ldots ,m\right)$$

(27) $$S_{i}^{-}=\alpha D_{i}^{-}+\beta R_{i}^{+}\left(i=1,2,\ldots ,m\right)$$

where α+β=1. Generally, α=β=0.5 is employed. $S_{i}^{+}$ and $S_{i}^{-}$ represent the proximity of the ith alternative to the positive and negative ideal solutions, respectively.

(8) Calculate the relative closeness ξ of the evaluation object, which can comprehensively reflect the proximity of the evaluation object to the ideal solution, as follows:

(28) $$\xi =\frac{S_{i}^{-}}{S_{i}^{-}+S_{i}^{+}}\left(i=1,2,\ldots ,m\right)$$

(9) Calculate the ranking of the evaluation objects.

According to the calculation result of Equation (28), the magnitude of the relative closeness ξ is used as the comprehensive evaluation value of the scheme evaluation. The larger the relative closeness ξ, the better the evaluation scheme.

## 4. Empirical Analysis

### 4.1. Data Source and Processing

#### 4.1.1. Data Source

In this study, the data from 30 provinces in China were selected for evaluation (Tibet, Taiwan, and other regions were not included, because data from these regions were not available). Data were obtained from the China Statistical Yearbook, China Logistics Yearbook, China Energy Statistical Yearbook, and statistical yearbooks of the selected provinces. Missing data were filled by linear interpolation, and data from the transportation, storage, and postal industries were selected to represent the logistics industry, as it occupies more than 85% of the output value.

#### 4.1.2. Measurement of the Carbon Emission Indicator in the Logistics Industry

Green logistics innovation under the background of “emission peak and carbon neutrality” should consider the carbon dioxide emissions of each province. Therefore, carbon emissions were introduced as a “non-expected output” index to measure the green and low-carbon development status of each province. Based on the statistical data of the transportation, storage, and postal industry from 2014–2018 China Energy Statistical Yearbook, the carbon emissions of the provincial logistics industry were measured by combining the carbon emission coefficient with eight types of energy consumption: raw coal, coking coal, gasoline, kerosene, diesel fuel, fuel oil, liquefied petroleum gas, and natural gas. The formula is expressed as follows:

(29) $$C=\sum_{i=1}^{8}E_{i}K_{i}$$

where *C* is the total carbon emissions, $E_{i}$ is the consumption of energy source *i*, and $K_{i}$ is the carbon emission coefficient of energy source *i*. The formula for $K_{i}$ is expressed as follows:

(30) $$K_{i}=SC_{i}\times CF_{i}\times SF_{i}\times \frac{44}{12}$$

where $SC_{i}$ is the average low-heating value of the energy source *i*, $CF_{i}$ is the carbon content per unit calorific value of the energy source *i*, $SF_{i}$ is the carbon oxidation rate of the energy source *i*, and 44/12 is the molecular weight of $CO_{2}$. In this study, $SC_{i}$ was retrieved from Appendix 4 of the China Energy Statistical Yearbook, and $CF_{i}$ and $SF_{i}$ were retrieved from the Provincial Greenhouse Gas Inventory Preparation Guidelines [47,48]. The relevant data on the carbon emission coefficients are listed in Table 3.

### 4.2. Application of the Evaluation Methods Suggested

#### 4.2.1. Application of the Combination Weighting Method Based on the Game Theory

The index data for 2017 were selected as an example to measure the subjective, objective, and combination weights. The calculated weighting coefficients for the subjective and objective weights were 0.242 and 0.758, respectively, and were used to calculate the combination weights. Thereafter, the main influencing factors on the RLGIL of the 30 provinces were comprehensively reflected by the combination weights.

**Figure 2:**
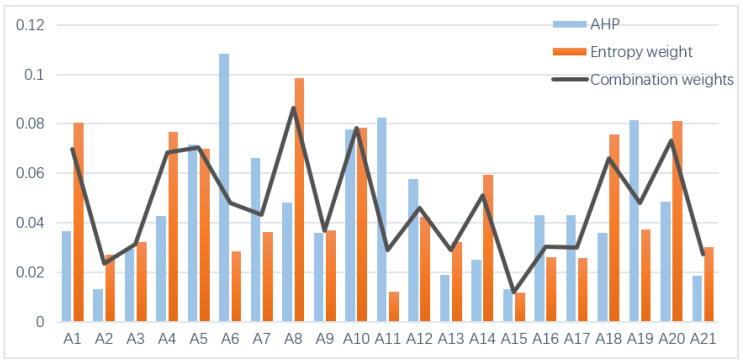
The subjective and objective index weights.

As shown in Figure 2, each index has a different impact on RLGIL. From the perspective of each criterion layer, the GIEL was identified as the most important factor affecting the RLGIL, followed by the GIIL and GIOL.

The GIEL was identified as the most important driving force in the logistics industry. Among its first-level indices, the logistics infrastructure level, and social development level were the most important, with weights of 0.1385 and 0.1745, respectively. A complete logistics infrastructure can help the logistics industry carry out green innovation activities to improve efficiency and reduce costs. Among the second-level indices, e-commerce sales had a remarkable impact on GIEL, with a weight of 0.0734. The logistics industry cannot be separated from the support of information technology to ensure service quality. The development of e-commerce provides a more convenient carrier for the innovative development of the logistics industry.

The GIIL had the second-highest impact on the RLGIL. Among its first-level indices, the weight of financial investment reached 0.1870, which indicates that financial investment is a necessary factor for the smooth development of logistics innovation activities. Human resources also play a key role in logistics innovation activities, which subsequently play an important role in the innovation process.

The GIOL had the lowest impact on the RLGIL. Among its first-level indices, the weight of the expected output reached 0.2447, which shows that the logistics industry attaches great importance to green innovation output. In the green innovation output, the impact of total postal business and the number of green patents granted was relatively large, with weights of 0.0863 and 0.0782, respectively. Technical patents provide an important basis for the development of innovation in the logistics industry, which also promotes the flow of innovation elements.

Among all second-level indices, the seven indices of R&D personnel full-time equivalent, R&D expenditure, local financial science and technology expenditure, number of green patents granted, total postal business, inland waterway mileage, and e-commerce sales accounted for 51.29% of the total. Thus, these seven indices are the most important basis for the RLGIL, which also triggers significant differences across regions.

#### 4.2.2. Application of the Evaluation Method Based on GRA-TOPSIS

In this study, a comprehensive evaluation was performed by constructing the GRA-TOPSIS model. First, a combination weighting method based on game theory was used to calculate the weight of the index system. Thereafter, the RLGIL of the 30 provinces was measured, where a higher value indicates a stronger green innovation level. Figure 2 shows the comprehensive evaluation results of RLGIL for the 30 provinces from 2013 to 2017.

**Figure 3:**
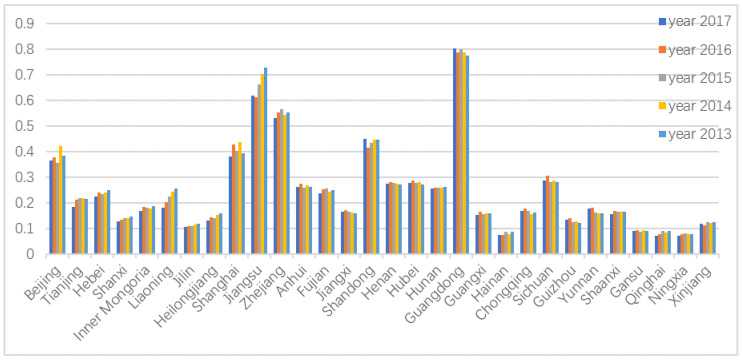
Comparison of the RLGIL comprehensive evaluation results.

As shown in Figure 3, the top four provinces in the 2017 ranking were Guangdong, Jiangsu, Zhejiang, and Shandong, whereas the four bottom provinces were Gansu, Hainan, Ningxia, and Qinghai. Thus, Figure 3 shows a clear gap between the RLGIL of the eastern and western regions. The Yangtze River Delta region, Beijing, and Guangzhou are relatively advanced in terms of their economic development. In fact, these regions invest sufficient funds in innovation and pay more attention to the green innovation environment. The rapid development of the logistics industry is also more attractive to talent, together with the inclination for financial investment, causing the RLGIL of these regions to be higher than that of the other regions. The average value for the 2017 RLGIL of the 30 provinces was 0.3728, and only 12 exceeded this value. Such a finding indicates that the RLGIL of many provinces still has room for improvement.

In terms of the time dimension, the ranking for the RLGIL from 2013 to 2017 did not change significantly, as most of the rankings fluctuated within a small range. The five provinces of Guangdong, Jiangsu, Zhejiang, Shandong, and Shanghai continuously occupied the top five positions, whereas the other provinces had a large gap. The RLGIL of Jiangsu has had a clear growth trend during the five years, gradually narrowing the gap with Guangdong. Returning to the original indices, the Jiangsu government was recognized to have increased investment in R&D, launched measures to accelerate the development of the modern logistics industry, strengthened technical support and R&D in science and technology, and promoted the development of green and low-carbon technologies. The RLGIL in Guangdong has a small decline, mainly because of high social logistics cost, low logistics operational efficiency, uneven regional development, and constrains of resources and environment.

**Figure 4:**
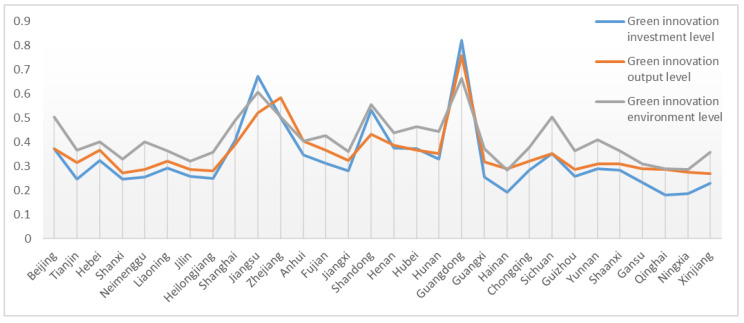
Comparison of the GIIL, GIOL, and GIEL.

Then, the three-criteria layer indices of the 30 provinces, GIIL, GIOL, and GIEL, were evaluated based on the 2017 index data. Figure 4 shows the criterion layer evaluation results for the 30 provinces in 2017.

As shown in Figure 4, large differences were found in the evaluation results of the GIIL, GIOL and GIEL. Guangdong ranked first for the comparison of the GIIL, GIOL, and GIEL, which highlights its unique advantage in all dimensions and a strong RLGIL. Jiangsu ranked higher than Zhejiang in innovation input but lagged behind in innovation output, indicating that Zhejiang has a higher output with lower input, which should be adopted by all provinces. Shanghai was equal to Beijing in the GIEL but had advantages in the GIIL and GIOL. As a result, Shanghai was generally ahead of Beijing. Jilin was slightly ahead of Heilongjiang in terms of innovation input and output, whereas Heilongjiang had a higher weight in GIEL. Therefore, Heilongjiang has an overall advantage over Jilin, as confirmed by the comprehensive evaluation results. Ningxia, Qinghai, and Hainan ranked the lowest for the three criterion layers. These provinces could take the following measures to improve RLGIL in multiple dimensions: increasing financial investment in green innovation R&D, enhancing the transformation rate of innovation achievements, and strengthening the construction of logistics infrastructure.

### 4.3. Spatial Effect Analysis on the RLGIL

#### 4.3.1. Spatial Distribution Characteristics of the RLGIL

**Figure 5:**
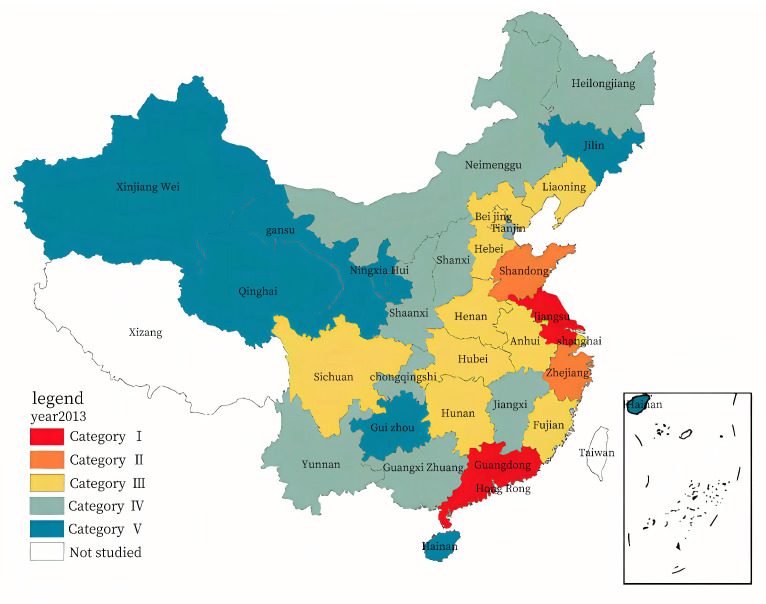
Spatial distribution for the RLGILs of the 30 provinces in 2013.

**Figure 6:**
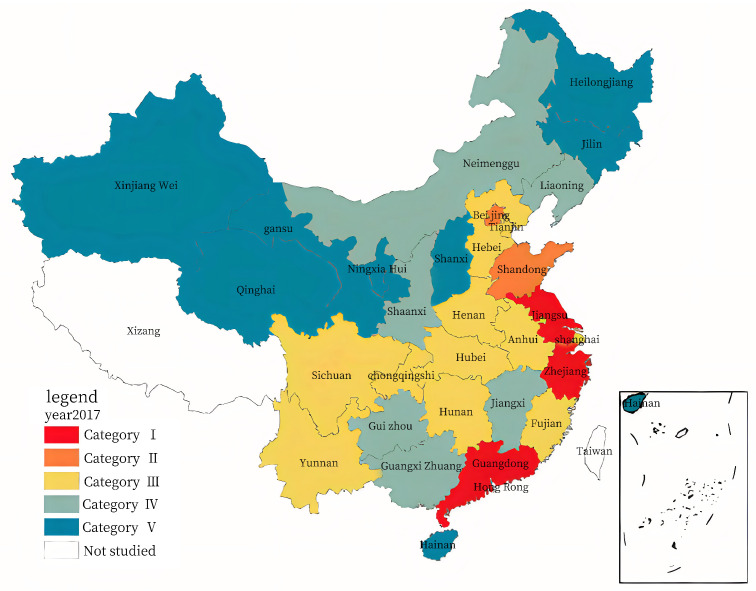
Spatial distribution for the RLGIL of the 30 provinces in 2017.

In terms of spatial-temporal evolution analysis, two years, 2013 and 2017, were selected for the comparative analysis. Using the ArcGIS10.8 software, the Jenks natural breaks classification method was used to classify the evaluation value for the RLGILs of the 30 provinces. Thereafter, the RLGIL for each year was divided into five categories to minimize the differences within each category and maximize the differences between categories. Figure 5 and Figure 6 show the spatial distribution maps.

As shown in Figure 5 and Figure 6, only four of the thirty provinces were listed in the first and second categories in 2013, increasing to six by 2017. There were ten provinces in the third category in 2013 and 2017. Nine of the thirty provinces were listed in the fourth category in 2013, which decreased to six by 2017. Seven of the thirty provinces were listed in the fifth category in 2013, which increased to eight by 2017.

The spatial distribution for the RLGIL of the 30 provinces exhibited the characteristics of “high in the east and south, low in the west and north.” The RLGIL of the eastern region was significantly higher than that of the midwest region, and the gap between the midwest provinces was smaller. The spatial distribution decreased from east to west; however, the overall development trend was smooth [49]. Moreover, the eastern region had a strong economic foundation, advanced technology, and a good innovation culture; therefore, this region had a better environment for green innovation.

The eastern provinces in the Yangtze River Delta and Pearl River Delta regions maintained high RLGILs. Guangdong and Jiangsu were among the provinces with extremely high RLGILs. Moreover, Zhejiang successfully ranked first, owing to five years of comprehensive development, gradually narrowing the gap between Guangdong and Jiangsu. Recalling the indices, the three provinces continued to increase their investment in R&D and e-commerce, promoting RLGIL. As a pioneer in reform, Guangdong had inherent advantages in talent attraction, policy, and financial fund inclination, which can provide strong support for enterprises to implement green innovation. As the second largest economic province in China, Jiangsu has always been at the forefront of green logistics innovation. Zhejiang, Shandong, and Shanghai also maintained high RLGIL, relying on the strong impetus of the Yangtze River Delta.

The midwest and northeast provinces had a lower level or even decreasing RLGIL, which might be mainly due to the following reasons: special geographical location, slow development of the logistics industry, low investment in infrastructure, lower technological human resources, advanced production technology relative to that in the east, and insufficient vitality of logistics green innovation.

#### 4.3.2. Global and Local Correlation Tests on the RLGIL

Using Geoda software, the spatial association characteristics for the RLGILs of the 30 Chinese provinces were analyzed by calculating the spatial weight matrix with rook adjacency and measuring the global Moran’s I index [50]. The statistical test results of Moran’s I Index for the RLGIL of the 30 provinces from 2013 to 2017 were obtained using 999 random permutations, as shown in Table 4.

As shown in Table 4, Moran’s I index for the RLGIL was significantly greater than 0, with all P values greater than 0.0824. This result indicates a significance at the 0.1 confidence level and a high positive spatial correlation in the study area, ultimately demonstrating that provinces with similar RLGILs show a certain agglomeration situation.

**Figure 7:**
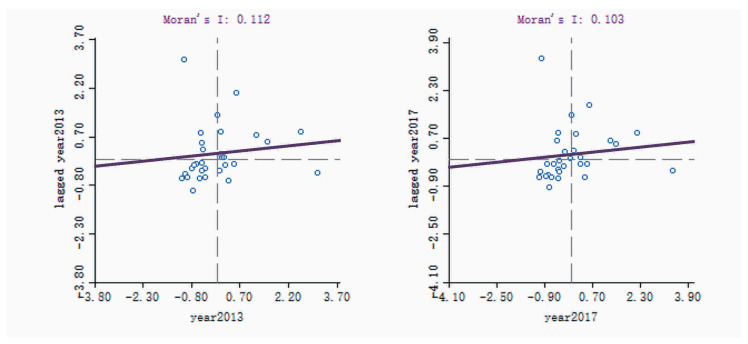
Moran’s I index scatter charts of the RLGIL in 2013 and 2017.

A local Moran’s I index scatter analysis was conducted to further examine the spatial dependence of the RLGIL. Moran’s I index scatter charts of RLGIL in 2013 and 2017 are shown in Figure 7. The horizontal axis represents logistics green innovation, and the vertical axis represents the spatial lag of logistics green innovation.

As depicted in Figure 7, the provinces in the first quadrant (H−H) in 2013 were Jiangsu, Zhejiang, Shanghai, Shandong, Anhui, Hunan, Hebei, and Henan (i.e., a total of eight provinces), accounting for 26.6% of the examined provinces. There were 12 provinces in the third quadrant (L−L), accounting for 40% of the total. The provinces in the second quadrant (L−H) were Hainan, Fujian, Jiangxi, Guangxi, and Tianjin (i.e., a total of five provinces), accounting for 16.6%. The provinces in the fourth quadrant (H−L) were Guangdong, Beijing, Hubei, Liaoning, and Sichuan (i.e., a total of five provinces).

In 2017, the number of provinces located in the first and third quadrants showed little change, 8 and 13, respectively, accounting for 70% of all provinces examined. In particular, Fujian jumped from the second quadrant (L−H) to the first quadrant (H−H), Liaoning moved from the fourth quadrant (H−L) to the third quadrant (L−L), and the remaining provinces located in the second and fourth quadrants remained unchanged.

In summary, the spatial pattern for the RLGIL of the 30 provinces was relatively stable, and the positions of most provinces remained unchanged. The provinces in the high-value agglomeration area (H−H) were few and were mainly concentrated in the eastern coastal region. The provinces in the low-value agglomeration area (L−L) were most concentrated in the midwest and northeast regions. The unbalanced distribution for RLGIL in the 30 provinces was serious, thereby aligning with the results from the previous section. Such results also show that a spatial agglomeration effect of logistics green innovation exists in the 30 provinces; however, the difference in the RLGIL between provinces still exists in the five years, and narrowing the gap is difficult to achieve in a short time.

## 5. Conclusions

The development level of regional logistics green innovation is an important standard for measuring the competitive strength of the regional logistics industry and an important factor in promoting regional economic green development. In this study, a comprehensive evaluation index system was constructed for the RLGIL using the VIF variance coefficient method to evaluate 30 provinces in China from 2013 to 2017. In addition, a combination weighting method based on the game theory was adopted to assign weights to the indices. Thereafter, the GRA-TOPSIS method was employed to perform a comprehensive evaluation, and a spatial effect analysis was conducted based on the Jenks natural breaks classification method and Moran’s I index. The following conclusions were drawn from the obtained results:

(1) The seven indices of R&D personnel full-time equivalent, R&D expenditure, local financial science and technology expenditure, number of green patents granted, total postal business, inland waterway mileage, and e-commerce sales were the most important factors affecting the RLGIL, and were significant reasons for the differences across regions.

(2) The RLGIL of the 30 provinces of China was clearly differentiated between east and west, where uneven development was identified. Provinces such as Guangdong, Jiangsu, and Zhejiang, had stronger RLGILs, whereas others had a large gap. The RLGIL of Jiangsu displayed a significant growth trend over the five years, gradually narrowing the gap with that of Guangdong. Most of the remaining provinces fluctuated within the stable range of the rankings. Differences were found between the evaluation results for each criterion layer. Relatively low innovation input should be used to obtain high innovation output, and the RLGIL should be improved from multiple dimensions.

(3) In terms of spatial distribution, the results revealed a high positive spatial correlation among the RLGIL of the 30 provinces. Further, the overall spatial distribution displayed a characteristic of “high in the east and south, and low in the west and north”. The RLGIL significantly varied among provinces, but displayed an overall trend of steady development. Regarding spatial agglomeration, a spatial agglomeration effect was identified in the RLGIL of the 30 provinces. The high-value aggregation areas were mainly concentrated in the eastern coastal region, and the low-value aggregation areas were in the midwest and northeast regions. Such a finding indicates a serious imbalance in the RLGIL in China’s regions.

## 6. Promotion Strategies

Based on the findings of this study, several promotion strategies were proposed:

(1) The government should improve the logistics green innovation input system and enhance the level of green innovation inputs. Further, talents and funds are necessary to ensure the stable development of logistics enterprises. Accordingly, logistics enterprises need to attract more talent by increasing their investments. Simultaneously, logistics enterprises can conduct co-cultivation with universities, and universities can train talent according to their actual needs; technological achievements can be applied to innovation in the logistics industry in reverse. In addition, the government can introduce corresponding policies and financial support to reduce pressure on enterprise financing and encourage green technological innovation. The government has the responsibility and ability to enact stricter environmental regulations and guide logistics enterprises to implement green innovation and increase tax preferences and financial subsidies.

(2) The government should establish a complete green innovation output system and improve the level of green innovation output. The boundaries of green logistics innovation between departments, industries, and regions should be removed by formulating regulations and strengthening the participation of capital investment from all walks of life. Meanwhile, logistics enterprises should respond to the government’s call to actively invest in innovative logistics construction, improve the conversion rate of innovation achievements, and transform various innovation resources into innovation outputs. The government could also encourage enterprises to use energy-saving and environmentally friendly technologies to reduce the overall energy consumption and pollutant emissions of the logistics industry. Active research on low-carbon green technologies can effectively reduce carbon emissions, enhance enterprises’ core competitiveness, and improve the contribution of logistics enterprises to regional green innovation.

(3) The government should accelerate the construction of logistics infrastructure; help the logistics industry transform into green innovation and achieve a green innovation environment; improve the accuracy of investment in logistics infrastructure construction; improve the efficiency of infrastructure operation; compensate for economic shortcomings; and reduce logistics costs by ultimately increasing research on green innovation support policy; improving the environmental protection governance regulatory system; continuously increasing financial expenditure on regional infrastructure development, energy conservation, governance, and construction; building a fair and balanced policy environment for green innovation; promoting a balanced development; completing the construction of an information-sharing platform for the logistics industry; improving informatization construction; strengthening investment in logistics infrastructure; optimizing resource allocation; and fully playing its key role.

## 7. Research Limits and Further Directions of Investigation

Investigating regional logistics levels in 30 provinces in China is a complex research activity that requires multiple approaches from the perspective of the designed evaluation index system and the statistical data used to design the research model. The authors acknowledge that there are some study limitations determined by the methods employed and the datasets used. For the future, it is possible to expand the dataset used, pick a different evaluation model, and improve the method by adding new components, potentially improving results and reducing limitations. At the same time, we can choose a micro perspective to study the green innovation level of logistics enterprises.

## Figures and Tables

**Table 1:** Regional logistics green innovation level evaluation index primary system.

Criterion Layer	First-Level Index	Second-Level Index
Green innovation input level (GIIL)	Human resources input	R&D personnel full-time equivalent
		Logistics industry employees
		Number of college students
		Number of R&D personnel in colleges and universities
		Number of R&D personnel in enterprises above scale
		Full-time equivalent of R&D personnel in enterprises above scale
		Number of scientific and technological personnel
		Number of scientific and technological personnel in scientific research institutions
	Financial funds input	R&D expenditure
		S&T activities funding
		R&D expenditure of colleges and universities
		Local financial science and technology expenditure
		R&D expenditure of scientific research institutions
		Total expenditure on transport
		Expenditure on developing new products for industrial enterprises above scale
		Proportion of fiscal expenditure on science and technology to GDP
Green innovation output level (GIOL)	Expected output	Value added of logistics industry
		Total postal services
		Total telecommunication services
		Freight volume
		Freight turnover
		Passenger volume
		Railway passenger volume
		Highway passenger volume
		Express volume
		Number of green patents granted
		Technology market turnover
	Non-expected output	Carbon dioxide emissions
		Industrial wastewater emissions
		Sulfur dioxide emissions
		Nitrogen oxide emissions
		Flue dust emissions
		Petroleum emissions
Green innovation environment level (GIEL)	Social development level	Regional GDP
		GDP per capita
		Number of permanent populations
		Resident consumption level
		Urban road area per capita
		Park green area per capita
		Retail sales of social consumer goods
		Per capita disposable income
		Urban green area, forest coverage
		Greening coverage of built-up areas
	Logistics infrastructure level	Highway mileage
		Railroad mileage
		High-speed grade highway mileage
		Inland waterway mileage
		Fixed asset investment in logistics industry
		Port cargo throughput
		Highway operating vehicle ownership
	Informational development level	E-commerce sales
		E-commerce purchase amount
		Internet penetration rate
		Mobile subscription
		Number of internet users
		Internet broadband access users
		Number of computers per 100 people
		Length of long-distance optical cable lines
		Information technology services income

**Table 2:** Evaluation index system of regional logistics green innovation level.

Target Layer	Criterion Layer	First-Level Index	Second-Level Index	Unit
Regional logistics green innovation level (RLGIL)	Green innovation input level (GIIL)	Human resources input	R&D personnel full-time equivalent (A1)	Person year
			Number of college students (A2)	Million people
			Logistics industry employees (A3)	Million people
		Financial funds input	R&D expenditure (A4)	Million CNY
			Local financial science and technology expenditure (A5)	Billion CNY
			Total expenditure on transport (A6)	Billion CNY
	Green innovation output level (GIOL)	Expected output	Value added of logistics industry (A7)	Billion CNY
			Total postal business (A8)	Billion CNY
			Freight volume (A9)	Million tons
			Number of green patents granted (A10)	Item
		Non-expected output	Carbon dioxide emissions (A11)	104 tn
	Green innovation environment level (GIEL)	Social development level	Regional GDP (A12)	Billion CNY
			Number of permanent population (A13)	Million people
			Resident consumption level (A14)	Yuan
			Urban road area per capita (A15)	Square meter
		Logistics infrastructure level	Highway mileage (A16)	Kilometer
			Railroad mileage (A17)	Kilometer
			Inland waterway mileage (A18)	Kilometer
			Fixed asset investment in logistics industry (A19)	Billion CNY
		Informational development level	E-commerce sales (A20)	Billion CNY
			Internet penetration rate (A21)	Percentage

**Table 3:** Carbon emission coefficient of the eight energy sources.

Energy Sources	${\mathrm{SC}}_{i}$ (kJ/kg or m${}^{3}$)	${\mathrm{SF}}_{i}$ (T/TJ)	${\mathrm{SF}}_{i}$ (%)	$K_{i}$ (kgCO${}_{2}$/kg or m${}^{3}$)
Raw coal	20,908	26.37	0.94	1.9002
Coking coal	28,435	29.5	0.93	2.8604
Gasoline	43,070	18.9	0.98	2.9251
Kerosene	43,070	19.6	0.98	3.0334
Diesel fuel	42,652	20.2	0.98	3.0959
Fuel oil	41,816	21.1	0.98	3.1705
Liquefied petroleum gas	50,179	17.2	0.98	3.1013
Natural gas	38,179	15.3	0.99	0.2162

**Table 4:** Moran’s I index for the RLGILs of the 30 provinces.

Year	Moran	E(I)	Sd(I)	Z-Value	*p*-Value
2013	0.1120	−0.0345	0.1133	1.3540	0.0840
2014	0.1250	−0.0345	0.1094	1.5358	0.0750
2015	0.1340	−0.0345	0.1087	1.6207	0.0680
2016	0.1040	−0.0345	0.1087	1.3111	0.0950
2017	0.1030	−0.0345	0.1051	1.3736	0.0900

## Data Availability

Not applicable.
